# Neurodevelopmental Alterations in Inhibitory Interneurons of Cortex, Hippocampus, and Striatum of Genetic Absence Epilepsy Rats

**DOI:** 10.1002/cns.71110

**Published:** 2026-09-03

**Authors:** Nihan Çarçak, Elif Tuğçe Erdeve, Courtney J. Wright, Elif Keskinöz, My Andersson, Filiz Onat, Deniz Kırık

**Affiliations:** ^1^ Department of Pharmacology, Faculty of Pharmacy Istanbul University Istanbul Türkiye; ^2^ Department of Neuroscience, Graduate School of Health Sciences Acibadem Mehmet Ali Aydinlar University Istanbul Turkey; ^3^ Department of Pharmacology, Institute of Graduate Studies in Health Sciences Istanbul University Istanbul Türkiye; ^4^ BRAINS Unit, BMC D11, Department of Experimental Medical Science Lund University Lund Sweden; ^5^ Department of Anatomy, School of Medicine Acibadem Mehmet Ali Aydinlar University Istanbul Türkiye; ^6^ Department of Medical Pharmacology, School of Medicine Acibadem Mehmet Ali Aydinlar University Istanbul Türkiye

**Keywords:** cortical development, epileptogenesis, GAERS, inhibitory interneuron, neuroscience, parvalbumin, postnatal development, somatostatin, spike‐and‐wave discharges (SWDs)

## Abstract

**Aims:**

Absence seizures, characterized by spike‐and‐wave discharges (SWDs), are mediated by reciprocal thalamocortical interactions; however, the contribution of developing inhibitory networks to SWDs remains unclear. We investigated the developmental trajectory of inhibitory interneurons in Genetic Absence Epilepsy Rats from Strasbourg (GAERS) by analyzing their distribution across postnatal development in the somatosensory (S1) and motor (M1) cortices, the hippocampus, and striatum.

**Methods:**

The neurodevelopmental trajectory of parvalbumin‐positive (PV+) and somatostatin‐positive (SST+) interneurons was quantified at three critical stages: postnatal day 14 (P14), when SWDs were not yet observed, P21 when immature SWDs appear, and adulthood (P90), when mature SWDs are established. Wistar rats served as controls. Brain sections were processed immunohistochemically to quantify interneuron density.

**Results:**

PV+ interneuron density across S1 and M1 was significantly higher in GAERS at P14 than control. However, this difference was not maintained at P21 and adults. Conversely, SST+ interneurons exhibited a delayed increase in M1. GAERS displayed higher PV+ interneuron density in the dentate gyrus and CA1 at P14, whereas SST+ interneuron density remained unchanged across hippocampal subfields. Striatal PV+ and SST+ interneurons increased at later developmental stages, suggesting altered inhibition in basal ganglia.

**Conclusion:**

These findings demonstrate a temporally dynamic and region‐specific reorganization of interneurons in GAERS that may underlie absence epileptogenesis.

## Graphical abstract

Schematic conceptual model illustrating the proposed temporally distinct contributions of cortical interneuron populations to absence seizure maturation in GAERS shown within thalamocortical loop (VPM–TRN–cortex). Early PV+ interneuron upregulation primes cortical networks for SWD initiation, while later SST+ interneuron increases in M1 cortex contribute to seizure propagation and maintenance. At P14, when SWDs are not yet observed, early upregulation of PV+ interneurons (blue) in S1 and M1 cortices strengthens FFI, priming corticothalamic circuits for SWD initiation. By P21, as SWDs begin to emerge, PV+‐driven FFI remains prominent, while SST+ interneuron (orange) inputs start to increase. In adulthood, mature SWDs coincide with a marked enhancement of SST+ interneuron recruitment in the cortex, contributing to sustained inhibitory control that shapes seizure propagation and maintenance. Upward arrows indicate increased PV+ and SST+ interneuron density observed in the present study. The PV+ and SST+ interneuron interaction and the thalamocortical circuitry, including the TRN, are shown schematically based on previously established circuit organization and are intended to provide mechanistic context rather than represent direct observations from the present study. The red triangle denotes a corticothalamic pyramidal neuron, and the green circle indicates a thalamocortical relay neuron. Abbreviations: FFI, feed‐forward inhibition; L, cortical layer; M1, primary motor cortex; PV+, parvalbumin‐positive interneuron; S1, primary somatosensory cortex; SST+, somatostatin‐positive interneuron; TRN, thalamic reticular nucleus; VPM, ventral posteromedial thalamic nucleus. Created in BioRender. Onat, F. (2026) https://BioRender.com/irftlb9.

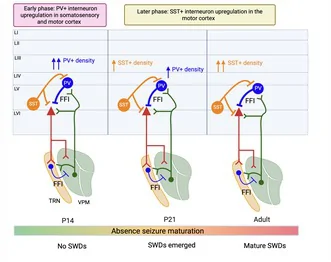

## 1 Introduction

Absence seizures, characterized by transient interruptions of consciousness, peri‐oral automatisms, and generalized spike‐and‐wave discharges (SWDs) on EEG, represent one of the most prevalent phenotypic manifestations of genetic generalized epilepsies. These seizures arise from abnormal oscillatory activity within the cortico‐thalamo‐cortical (CTC) network [1, 2], where an imbalance between excitatory and inhibitory transmission is thought to be a critical trigger [3, 4]. Disruptions in inhibitory control have been implicated in several monogenic and inbred models of absence epilepsy [5, 6, 7]. Furthermore, human genetic studies identifying mutations in *γ*‐aminobutyric acid (GABA) receptor subunits underscore the clinical relevance of GABAergic interneuron dysfunction in generalized seizures [8]. Together, these findings indicate that impaired inhibitory interneuron function may destabilize CTC oscillations and contribute to the epileptogenesis of absence seizures.

Among neocortical GABAergic interneurons, the two predominant populations are parvalbumin‐expressing (PV+) fast‐spiking interneurons and somatostatin‐expressing (SST+) low‐threshold spiking interneurons. Both originate from the medial ganglionic eminence and express overlapping transcription factors that determine their laminar positioning and connectivity within the cortex [9, 10]. In the somatosensory cortex (S1), SST+ interneurons primarily target distal dendrites of pyramidal neurons, providing feedback inhibition, whereas PV+ interneurons receive strong thalamocortical input and mediate fast feedforward inhibition (FFI) onto pyramidal cells and other cortical targets [11]. These complementary inhibitory mechanisms are crucial for maintaining temporal precision and network stability within cortical circuits (see graphical abstract).

The Genetic Absence Epilepsy Rats from Strasbourg (GAERS) constitute a well‐established model of human absence epilepsy, exhibiting spontaneous and recurrent SWDs associated with behavioral arrest. In GAERS, inhibitory synaptic input from layers IV–V to layers II–III of S1 was found to be reduced by approximately 50% at postnatal day 15 (P15) compared with Wistar controls [12]. This coincides with the first appearance of abnormal oscillations during the second postnatal week, which progressively evolve into mature SWDs by P25–P30 [13, 14]. Given that PV+ interneuron maturation also takes place during this period and marks the onset of the somatosensory critical window, the reduction in inhibitory input suggests a potential developmental and functional deficit in PV+ interneuron in this model.

Recent imaging studies have further revealed a disease‐specific resting‐state network in juvenile GAERS that differs markedly from age‐matched non‐epileptic controls [15]. These findings highlight cortical network alterations as a defining feature of absence epileptogenesis, suggesting that impaired development of inhibitory circuits may weaken FFI, reduce cortical control over thalamic drive, and promote SWD generation. Supporting this view, previous reports have described functional abnormalities in inhibitory signaling within S1 in various genetic models of absence epilepsy [16, 17, 18]. However, altered inhibition in absence epilepsy is not necessarily limited to reduced inhibition. In contrast to focal epilepsies, experimental evidence indicates that absence seizures are frequently associated with increased GABAergic inhibition within thalamocortical circuits, where enhanced inhibition can facilitate hypersynchronous oscillations and SWDs [19]. Consistent with this concept, Crunelli and colleagues [20] demonstrated increased GABA immunoreactivity in the thalamic reticular nucleus and S1 of GAERS, suggesting an overall enhancement of inhibitory tone. However, this observation does not identify which interneuron subtypes account for the change, emphasizing the need to identify specific interneuron contributions to altered inhibitory balance. In particular, how inhibitory PV+ and SST+ interneurons mature during the onset of absence epilepsy have not been quantitatively mapped before in literature.

To address this gap, we examined the neurodevelopmental profile of PV+ and SST+ GABAergic interneurons in the S1, a key cortical area involved in the initiation of SWDs, across three critical developmental stages: P14 (prior to SWD onset), P21 (early appearance of immature SWDs), and adult P90 (chronic stage with established SWDs) [21]. Using immunocytochemistry, we quantified interneuron densities across superficial (LII–IV) and deep (LV–VI) layers of S1 and compared GAERS with age‐matched Wistar controls. To evaluate regional specificity, we also assessed interneuron distribution in the motor cortex (M1), which maintains relatively preserved intrinsic electrophysiological properties in GAERS [22], as well as in the hippocampal subfields: dentate gyrus (DG), CA1, CA3, and striatum, regions that modulate SWDs via cortico‐basal ganglia‐thalamic loops [23].

## 2 Methods

### 2.1 Animals

All animal experiments were conducted in accordance with the EU Directive 2010/63/EU for animal experiments and approved by the Institutional Animal Care and Use Committee of Acıbadem University (ethical approval number: 2022/82). Male and female GAERS and Wistar rats were used at P14 and P21, and an additional male‐only adult (P90) cohort was included in the present study (*n* = 6 animals per group). All rats were housed in home cages with temperature (20°C–25°C) and humidity (40%–60%) control in a facility under a 12‐h light/dark cycle with food and water provided ad libitum.

### 2.2 Tissue Collection and Processing

Rats were anesthetized with 3%–4% isoflurane and transcardially perfused using a peristaltic pump (Masterflex MFLX07522‐20, VWR International, USA) with 0.1 M phosphate‐buffered saline (PBS), followed by 4% paraformaldehyde (PFA) in PBS Merck on a ventilated perfusion table. Brains were carefully collected and post‐fixed in 4% PFA in PBS at 4°C overnight, then transferred to 25% sucrose solution for cryoprotection. 40 μm‐thick coronal sections were sectioned in 10 series using a freezing microtome (Microm HM 450, Thermo Scientific, USA) fitted with a home‐built cryo‐holder and stored in antifreeze solution until further processing. To ensure reproducibility and anatomical consistency across all subjects, sections from the same series number (representing identical or nearly identical stereotaxic coordinates) were selected for immunohistochemical staining and subsequent quantitative analysis.

### 2.3 Immunohistochemistry

Sections were thoroughly washed with tris buffered saline (TBS) and treated with a 3% hydrogen peroxide and 10% methanol solution to quench endogenous peroxidase activity. Blocking for non‐specific binding of the secondary antibodies was achieved by pre‐incubation of the sections in 5% normal horse serum in 0.25 Triton‐X in TBS (TBS‐T), prior to incubation in mouse monoclonal primary antisera directed against parvalbumin (1:32 000; MAB1572; Millipore Sigma; Darmstadt, Germany), or rabbit recombinant monoclonal somatostatin 28 antibody (1:4000; EPR3359 (2), ab111912; Abcam, Cambridge, UK) in TBS‐T containing 1% bovine serum albumin (BSA), overnight at room temperature. The next day, sections were incubated in biotinylated secondary immunoglobulins 1:200; horse anti mouse; BA‐2001; and 1:400; horse anti rabbit; BA1100 (both obtained from Vector Laboratories, USA for parvalbumin and somatostatin respectively) in 1% BSA/TBS‐T for an hour at room temperature. After subsequent washing, the sections were incubated in VECTASTAIN Elite ABC‐HRP kit (Vector Laboratories Inc., United States) for 1 h at room temperature and then washed with TBS. Peroxidase labelling was visualized with a chromogenic 3,3′‐diaminobenzidine (DAB) reaction resulting in a brown precipitate. The sections were mounted on chrome‐gelatine‐coated glass slides. After series of dehydration steps in ascending concentrations of ethanol (70%, 95%, 100%) and xylene they were cover slipped with DPX (Sigma Aldrich). Sections stained with parvalbumin antibody were additionally counterstained with cresyl‐violet as follows: dehydration in ascending ethanol concentrations (70%, 95%, and 100%), xylene, rehydration with descending ethanol concentrations (100%, 95%, 70%, and de‐ionized water) and cresyl‐violet followed by ascending ethanol series (70%, 95%, and 100%), cleared in xylene and then cover slipped with DPX.

### 2.4 Image Analysis

To ensure anatomical consistency across all animals, regions of interest (ROIs) were defined according to the Paxinos and Watson Rat Brain Atlas [24]. From each animal, to achieve a representative average, three sections containing the S1, M1, DG, CA1, and CA3 subregions of the hippocampus and striatum were captured with light microscopy (Carl Zeiss Primostar) using a Plan‐ACHROMAT 4X (NA 0.1) objective. For quantification of PV+ neurons, the Cell Counter plugin in Fiji software (version 1.54j) was used and neurons were counted manually [25]. This approach was selected because the high staining intensity of the PV+ neuropil and axonal plexuses created a low signal‐to‐background ratio that prevented accurate automated quantification. Quantification of SST+ neurons was automatized by using QuPath (v.0.5.1) [26]. Cell detection was performed using the Positive Cell Detection tool, with optical density sum as the quantification method, pixel size of 2 μm. Detection parameters included a sigma value of 3 μm and a threshold of 0.1. Only nuclei with an area between 120 and 400 μm^2^ were included in the analysis. ROIs were manually selected using the closed polygon annotation tool, and a standardized detection script including these parameters was applied to each ROI.

### 2.5 Statistical Analysis

Interneuron density (cells/mm^2^) was averaged across three technical replicate sections per animal for each brain region and cell type prior to statistical analysis. This approach was validated by comparing it against a linear mixed model incorporating the full replicate‐level dataset (1689 sections, 36 animals), which yielded nearly identical strain‐difference estimates (mean absolute difference = 0.35 cells/mm^2^ across 48 comparisons), confirming that averaging technical replicates did not bias the results. Prior to analysis, data were tested for normality using the Shapiro–Wilk test (Prism, version 10.4.2; GraphPad Software, San Diego, CA, USA), and outliers were identified using the ROUT method (Q = 1%) and are indicated in the figures as open circles but were not excluded from the analyses.

**FIGURE 1:**
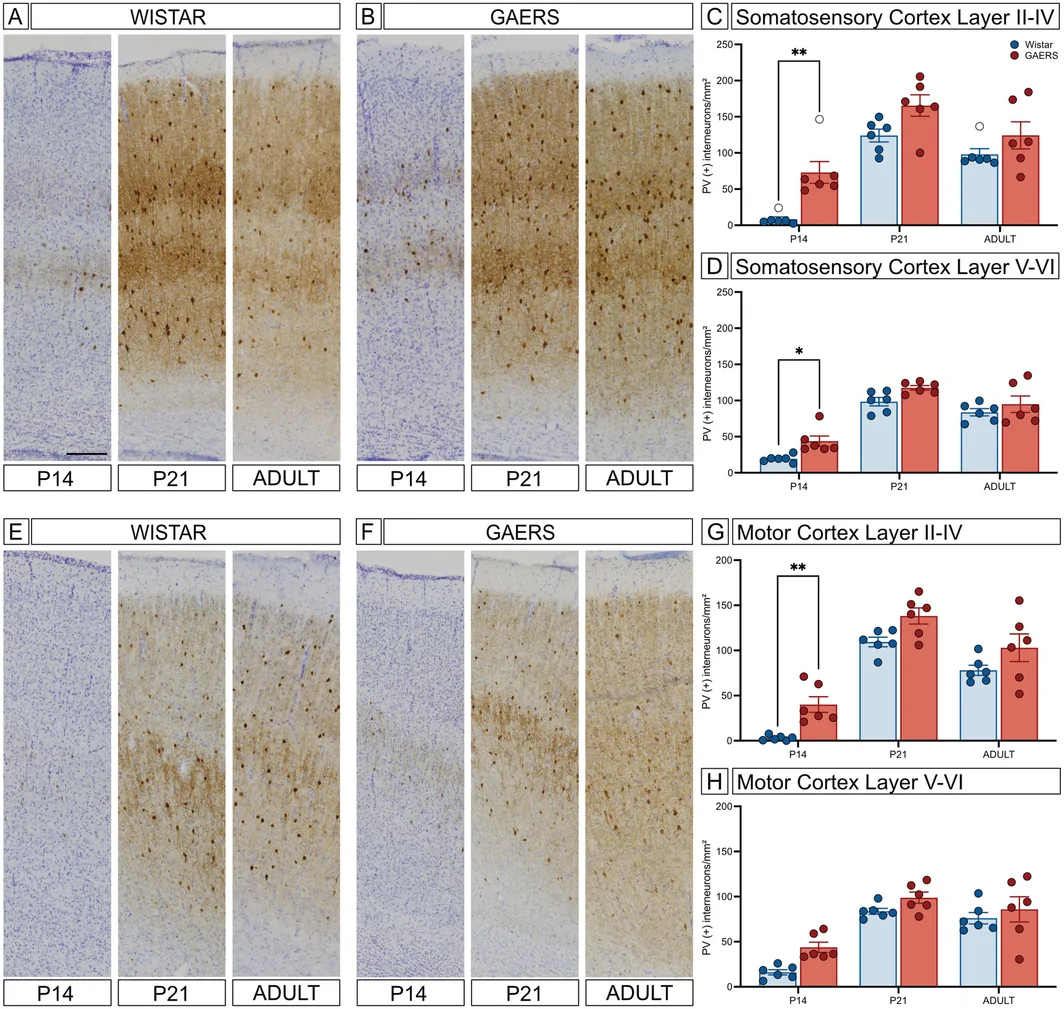
Developmental changes in PV+ interneuron density in the S1 and M1 cortices of Wistar and GAERS. Representative images of PV immunostaining in the S1 cortex of Wistar (A) and GAERS (B), and in the M1 cortex of Wistar rats (E) and GAERS (F) across three developmental stages: P14, P21, and adult (P90). Quantification of PV+ interneuron density (cells/mm^2^) in layers II–IV (C) and layers V–VI (D) of S1, and in layers II–IV (G) and layers V–VI (H) of M1. The circles showing for Wistar in blue; GAERS in red, represents individual animals (C, D, G and H) which correspons to the average density across the three slices analyzed per animal. Data are presented as mean ± SEM. Scale bar in A is 200 μm and applies to all panels. Statistical comparisons were performed using a linear mixed model with Strain and Age as fixed effects and a random intercept for animal, fit separately for each region/cell‐type panel; residual variance was allowed to differ by age where a likelihood ratio test indicated significant heteroscedasticity. Strain comparisons at each age were tested using estimated marginal means with Benjamini–Hochberg FDR correction applied across all 48 comparisons reported in Figures 1, 2, 3, 4 (*p < 0.05, **p < 0.01, ***p < 0.001, FDR‐corrected).

**FIGURE 2:**
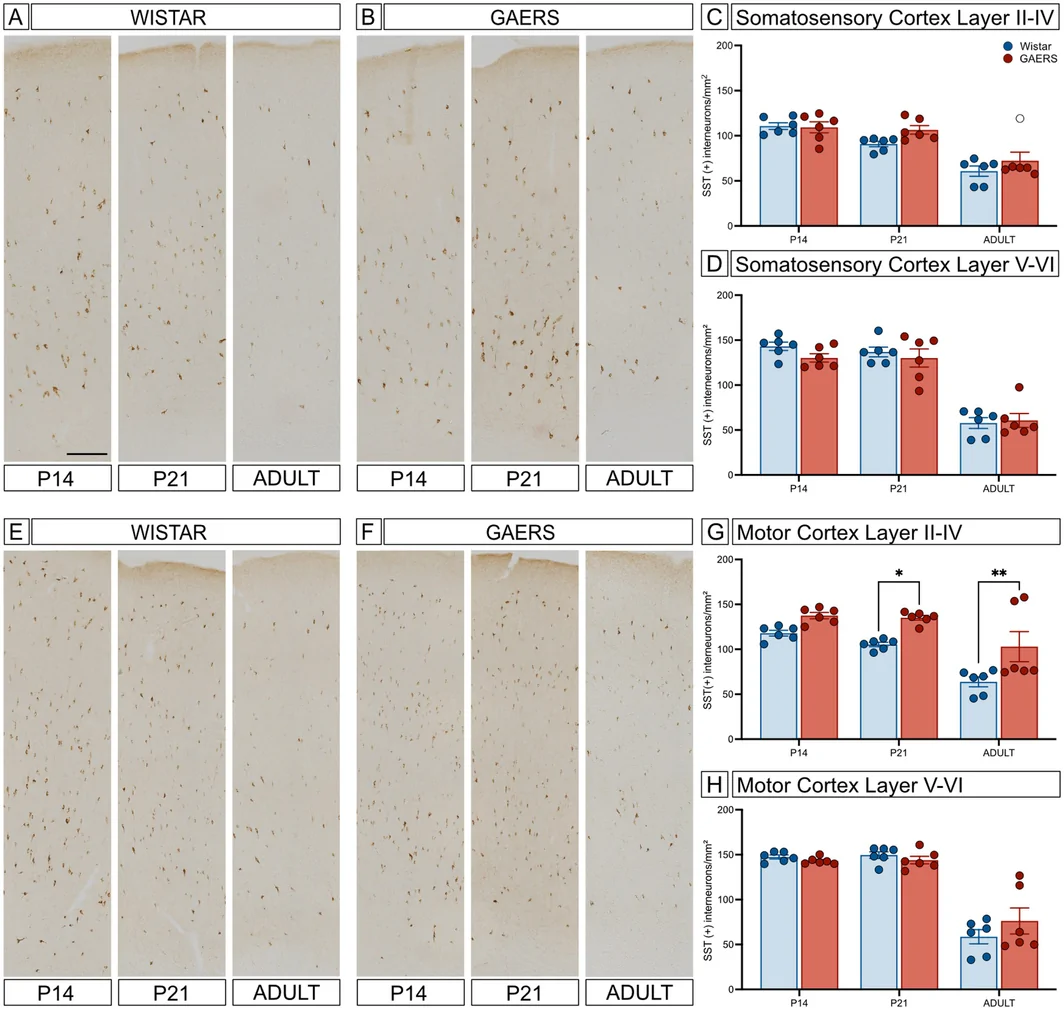
Developmental changes in SST+ interneuron density in the S1 and M1 cortices of Wistar and GAERS. Representative images of SST immunostaining in the S1 cortex of Wistar (A) and GAERS (B), and in the M1 cortex of Wistar (E) and GAERS (F) across three developmental stages: P14, P21, and adult (P90). Quantification of SST+ interneuron density (cells/mm^2^) in layers II–IV (C) and layers V–VI (D) of S1, and in layers II–IV (G) and layers V–VI (H) of M1. The circles showing for Wistar in blue; GAERS in red, represents individual animals (C, D, G and H) which corresponds to the average density across the three slices analyzed per animal. Data are presented as mean ± SEM. Scale bar in A is 200 μm and applies to all panels. Statistics as in Figure 1 (*n* = 6 animals/strain/age). Age‐heteroscedastic residual variance was used for S1 LII‐IV and S1 LV‐VI; M1 LII‐IV and M1 LV‐VI used homogeneous variance. *p < 0.05, **p < 0.01, ***p < 0.001 (FDR‐corrected).

**FIGURE 3:**
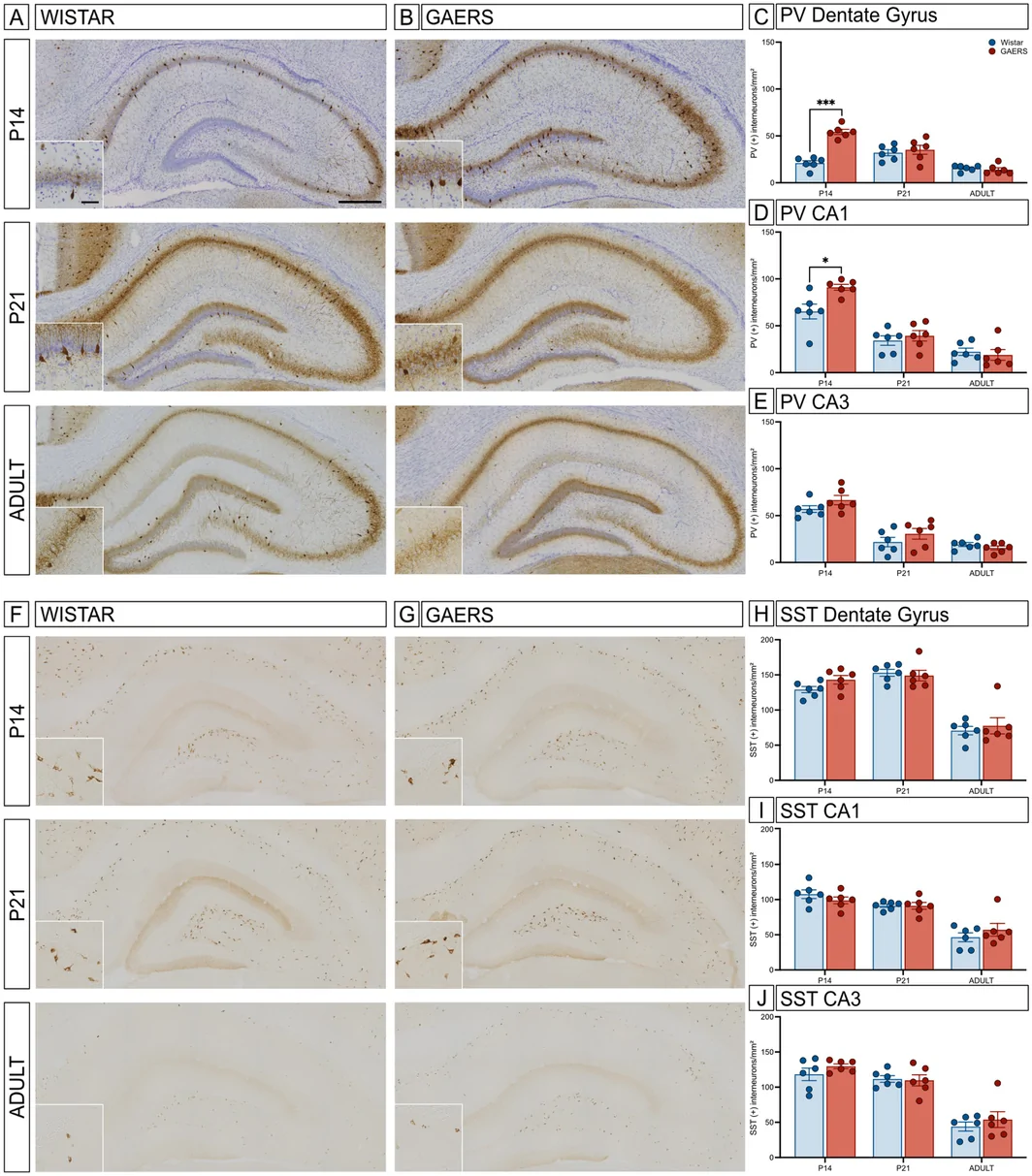
Developmental changes in PV+ and SST+ interneuron density in the hippocampal subfields (DG, CA1, CA3) of Wistar and GAERS. Representative images of PV immunostaining in the hippocampus of Wistar (A) and GAERS (B) at P14, P21, and adult (P90). (C–E) Quantification of PV+ interneuron density (cells/mm^2^) in the DG (C), CA1 (D), and CA3 (E) regions at the three developmental stages. Representative images of SST immunostaining in the hippocampus of Wistar (F) and GAERS (G) at P14, P21, and adult. Quantification of SST+ interneuron density (cells/mm^2^) in the DG (H), CA1 (I), and CA3 (J) regions across developmental stages. The circles showing for Wistar in blue; GAERS in red, represents individual animals (C, D, E, H, I and J) which corresponds to the average density across the three slices analyzed per animal. Data are presented as mean ± SEM. Scale bar in A is 500 μm and applies to all panels. Scale bar in higher magnification inset in the hippocampal panels h represent 50 μm. Statistics as in Figure 1 (*n* = 6 animals/strain/age). Age‐heteroscedastic residual variance was used for DG‐PV, DG‐SST, CA1‐PV, CA3‐PV, and CA3‐SST; CA1‐SST used homogeneous variance. *p < 0.05, **p < 0.01, ***p < 0.001 (FDR‐corrected).

**FIGURE 4:**
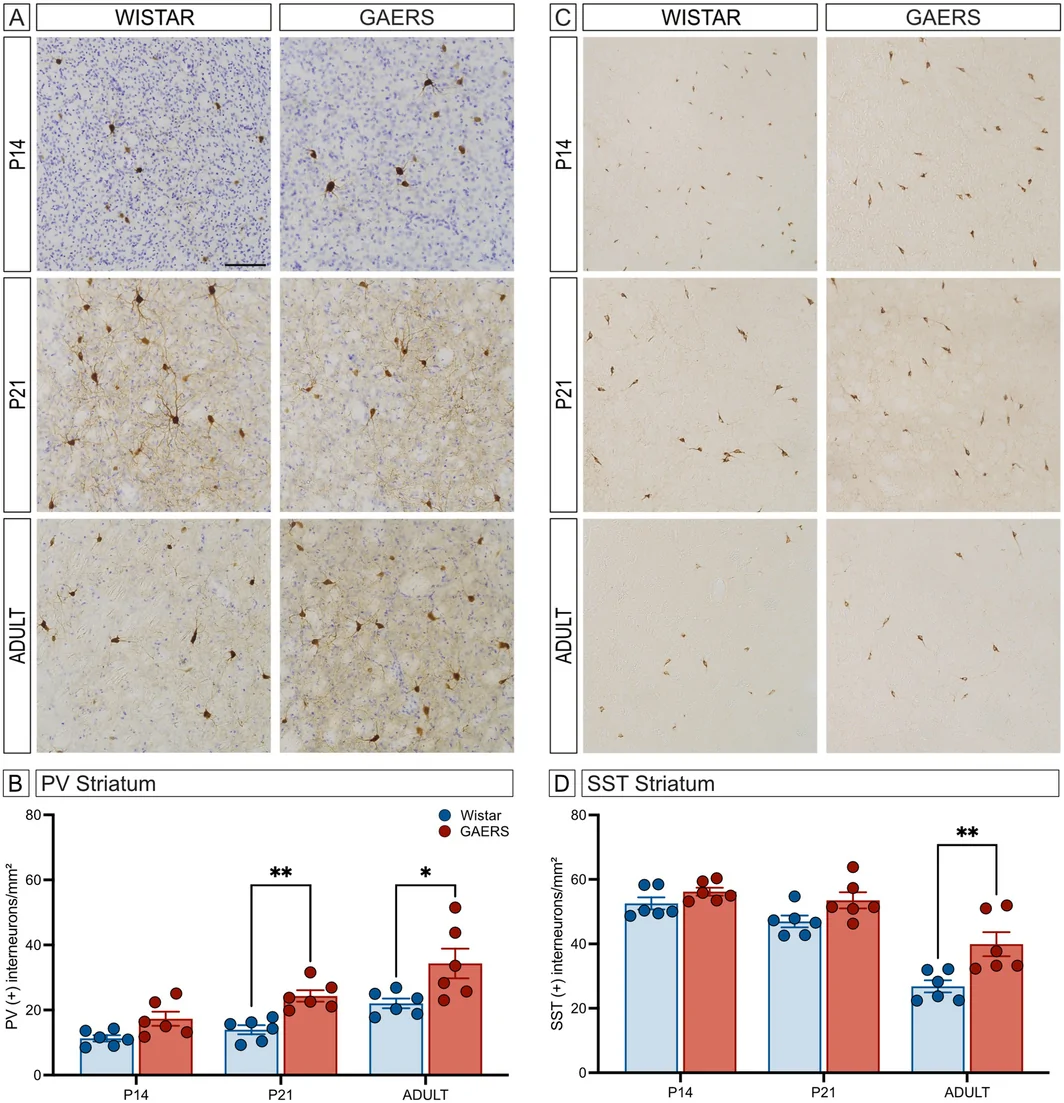
Developmental changes in PV+ and SST+ interneuron density in the striatum of Wistar and GAERS rats. Representative images of PV immunostaining in the striatum of Wistar and GAERS at P14, P21, and adult (P90). (A) Quantification of PV+ interneuron density (cells/mm^2^) in the striatum (B) at the three developmental stages. Representative images of SST immunostaining in the hippocampus of Wistar and GAERS rats at P14, P21, and adult (P90) (C). Quantification of SST+ interneuron density (cells/mm^2^) in the striatum (D) across developmental stages. The circles showing for Wistar in blue; GAERS in red, represents individual animals (B and D) which corresponds to the average density across the three slices analyzed per animal. Data are presented as mean ± SEM. Scale bar in A is 100 μm and applies to all panels. Statistics as in Figure 1 (*n* = 6 animals/strain/age). Age‐heteroscedastic residual variance was used for the PV panel; the SST panel used homogeneous variance. *p < 0.05, **p < 0.01, ***p < 0.001 (FDR‐corrected).

For each of the 16 Region × Cell type combinations, animal‐averaged density was analyzed using a linear mixed model with Strain and Age as fixed effects and a random intercept for animal, fit separately for each panel in R (version 4.3.3) using the nlme package. Homogeneity of residual variance across ages was formally tested within each panel by comparing a standard model against one allowing residual variance to differ by age, using a likelihood ratio test; the heteroscedastic model was adopted for panels showing significant age‐related heteroscedasticity (10 of 16 panels, all *p* < 0.05). Normality of model residuals was assessed using the Shapiro–Wilk test, residuals departed significantly from normality in 7 of 16 panels (predominantly SST datasets), partly attributable to two individual animals with elevated density across multiple regions (GAERS). A sensitivity analysis excluding these animals did not alter the significance conclusion for the strain main effect in any affected panel, indicating that the reported effects are not driven by non‐normality or influential observations. Post hoc comparisons of Strain at each Age were extracted using estimated marginal means (emmeans package, version 1.10.0) and corrected for multiple comparisons across all 48 tests reported in Figures 1, 2, 3, 4 using the Benjamini–Hochberg false discovery rate (FDR) procedure, replacing the Bonferroni correction applied to individual two‐level comparisons in earlier analyses, which does not adjust *p*‐values when only two groups are compared. Data are reported as mean ± SEM, and FDR‐corrected *p* < 0.05 was considered statistically significant.

## 3 Results

### 3.1 Early Transient Increase in PV+ Interneuron Density in Somatosensory and Motor Cortex of GAERS

To assess layer‐specific developmental changes, analyses were performed separately for LII–IV and LV–VI (Figure 1A,B), using a linear mixed model with Strain and Age as fixed effects and a random intercept for animal to account for the three technical replicate sections per region (Tables S1 and S2). In upper layers LII–IV of S1, PV+ interneuron density changed significantly across developmental stages (age: *F* (2, 30) = 12.88, *p* < 0.001) and differed significantly by strain overall (*F* (1, 30) = 12.79, *p* = 0.001). A significantly higher number of PV+ interneurons was found at P14 in GAERS compared to age‐matched Wistar rats (Figure 1C; estimated difference = 64.67 cells/mm^2^, FDR‐corrected *p* = 0.010) but did not reach significance at P21 (FDR‐corrected *p* = 0.103) or adulthood (FDR‐corrected *p* = 0.318). Due to the high variance and number of analyses (power 58%), an absence of effect is not conclusive.

Similarly, PV‐expression in LV–VI showed an age‐dependent increase (age: *F* (2, 30) = 41.91, *p* < 0.001) and greater PV+ density in GAERS compared to Wistar rats overall (strain: *F* (1, 30) = 8.52, *p* = 0.007). Post hoc analysis revealed that GAERS at P14 exhibited significantly higher PV+ interneuron density than age‐matched Wistar rats (Figure 1D; estimated difference = 24.39 cells/mm^2^, FDR‐corrected *p* = 0.035; representative images are shown in Figure 1A,B), with no significant strain differences at P21 (FDR‐corrected *p* = 0.098) or adulthood (FDR‐corrected *p* = 0.203). No Strain × Age interaction was found in either layer (LII–IV: *p* = 0.298; LV–VI: *p* = 0.790), indicating that the overall developmental trajectory of PV+ expression in S1 is similar between strains. These findings indicate a transient, early postnatal increase in PV+ interneuron density in the S1 cortex of GAERS, particularly in superficial cortical layers. As S1 has been shown to be critically involved in the generation of absence seizures, this may reflect early alterations in inhibitory circuit maturation associated with the epileptic phenotype.

To determine whether a similar trend extends beyond S1, we analyzed PV+ interneurons in M1 (Figure 1E,F). Consistent with S1, PV+ cell counts in M1 LII–IV and LV–VI increased with age and differed by strain (LII–IV: age *F* (2, 30) = 45.83, *p* < 0.001, strain *F* (1, 30) = 17.04, *p* < 0.001; LV–VI: age *F* (2, 30) = 15.18, *p* < 0.001, strain *F* (1, 30) = 7.13, *p* = 0.012). At P14, GAERS exhibited significantly higher PV+ cell density than Wistar rats in upper layers (Figure 1G; estimated difference = 37.21 cells/mm^2^, FDR‐corrected *p* = 0.005). In lower layers, the P14 difference (estimated 27.76 cells/mm^2^, raw *p* = 0.012) is not significant (FDR‐corrected *p* = 0.053) and is better described as a non‐significant trend (Figure 1H). No significant strain differences were observed at P21 or adulthood in either layer (all FDR‐corrected *p* ≥ 0.098).

### 3.2 Altered Developmental Dynamics of SST+ Interneurons in Motor but Not Somatosensory Cortex of GAERS

Quantification of SST+ interneurons in LII–IV of S1 showed that SST+ cell density declined over time in both strains (age: *F* (2, 30) = 11.59, *p* < 0.001). The overall strain main effect was not significant (*F* (1, 30) = 0.02, *p* = 0.891), and no significant pairwise strain differences were observed at any individual age (all FDR‐corrected *p* ≥ 0.318), consistent with a developmental trajectory that is similar between strains (Figure 2C). LV–VI showed the same declining pattern (age: *F* (2, 30) = 40.20, *p* < 0.001), with no significant strain differences at any developmental stage (strain: *F* (1, 30) = 1.96, *p* = 0.172; all FDR‐corrected *p* ≥ 0.098) (Figure 2D).

M1 showed a similar developmental profile to S1, with decreasing SST+ density over time (LII–IV: age *F* (2, 30) = 6.47, *p* = 0.005; LV–VI: age *F* (2, 30) = 29.19, *p* < 0.001). In contrast to S1, upper layers of M1 in GAERS showed elevated SST+ density with a strain effect approaching significance (*F* (1, 30) = 3.34, *p* = 0.078), driven by significant pairwise differences at P21 (Figure 2G; estimated difference = 29.79 cells/mm^2^, FDR‐corrected *p* = 0.045) and adulthood (estimated difference = 39.14 cells/mm^2^, FDR‐corrected *p* = 0.010), with no difference at P14 (FDR‐corrected *p* = 0.196), suggesting altered developmental dynamics of this interneuron subtype as seizures develop. Deep layers showed no significant strain differences at any stage (strain: *F* (1, 30) = 0.18, *p* = 0.672) (Figure 2H).

### 3.3 Developmental Changes in PV+ and SST+ Interneuron Density in Hippocampal Subfields of Wistar Rats and GAERS

To investigate whether developmental changes in PV+ and SST+ interneurons extend beyond the cortex in GAERS, we examined their neurodevelopmental profile in hippocampal subfields (DG, CA1, and CA3) across postnatal development. As illustrated by representative images (Figure 3A,B), GAERS exhibited a significantly higher density of PV+ interneurons in the DG at P14 compared to Wistar rats (Figure 3C; estimated difference = 33.27 cells/mm^2^, FDR‐corrected *p* < 0.001), with no differences at P21 or adulthood (FDR‐corrected *p* ≥ 0.714). A significant Strain × Age interaction was confirmed (*F* (2, 30) = 25.43, *p* < 0.001), consistent with a strain‐specific developmental pattern restricted to P14.

In the CA1 region, a significant main effect of age was found (*F* (2, 30) = 41.39, *p* < 0.001), together with a significant overall strain effect (*F* (1, 30) = 9.17, *p* = 0.005) and a significant Strain × Age interaction (*F* (2, 30) = 3.38, *p* = 0.047). At P14, GAERS PV+ interneuron density was significantly higher than Wistar (Figure 3D; estimated difference = 25.86 cells/mm^2^, FDR‐corrected *p* = 0.030). No differences were observed at P21 or adulthood (FDR‐corrected *p* ≥ 0.640). A similar developmental trajectory was observed in CA3, predominantly shaped by age (*F* (2, 30) = 40.81, *p* < 0.001), with no significant strain effect (*F* (1, 30) = 2.11, *p* = 0.157) or pairwise differences at any stage (Figure 3E; all FDR‐corrected *p* ≥ 0.344). SST+ interneuron density in the DG showed a significant main effect of age (*F* (2, 30) = 25.88, *p* < 0.001) with no strain differences at any time point (Figure 3H; strain: *F* (1, 30) = 1.31, *p* = 0.261).

In CA1, SST+ density also showed a significant main effect of age (*F* (2, 30) = 14.08, *p* < 0.001) with no significant strain effect or interaction, and no significant pairwise differences (Figure 3I; strain: *F* (1, 30) = 1.10, *p* = 0.302). These findings suggest that SST+ interneuron development in CA1 follows a similar temporal pattern in both Wistar rats and GAERS, with age being the primary determinant of cell density changes. Similarly, in CA3, SST+ density varied significantly with age (*F* (2, 30) = 25.85, *p* < 0.001), with no significant strain differences at any stage (Figure 3J; strain: *F* (1, 30) = 0.92, *p* = 0.345).

### 3.4 Increased PV+ and SST+ Interneuron Density in Striatum of GAERS

As shown in representative images of striatal PV+ staining (Figure 4A), GAERS exhibited a persistent increase in PV+ interneuron density in the striatum, with a significant overall strain effect (*F* (1, 30) = 5.40, *p* = 0.027) and age effect (*F* (2, 30) = 14.64, *p* < 0.001). This difference was not significant at P14 (FDR‐corrected *p* = 0.098) and reached significance at P21 (Figure 4B; estimated difference = 10.11 cells/mm^2^, FDR‐corrected *p* = 0.005) and adulthood (estimated difference = 12.26 cells/mm^2^, FDR‐corrected *p* = 0.014), indicating altered inhibitory regulation in basal ganglia circuits, potentially contributing to the pathophysiology of absence epilepsy.

Representative images of striatal SST+ staining (Figure 4C) show a similar pattern, with GAERS exhibiting higher SST+ interneuron density compared to Wistar rats (Figure 4D). A significant main effect of age was found (*F* (2, 30) = 14.31, *p* < 0.001), but no significant overall strain effect (*F* (1, 30) = 1.24, *p* = 0.273); the strain difference reached significance only in adulthood (Figure 4F; estimated difference = 13.08 cells/mm^2^, FDR‐corrected *p* = 0.005), with no significant differences at P14 or P21 (FDR‐corrected *p* ≥ 0.160). The increased SST+ interneuron density in the striatum of GAERS in adulthood may reflect maladaptive inhibitory reorganization associated with the epileptic network. These findings suggest that absence epilepsy involves persistent alterations in GABAergic microcircuitry beyond the cortex and thalamus, potentially implicating the basal ganglia in the pathophysiology of SWDs.

## 4 Discussion

Although previous studies have reported alterations in the density of GABAergic interneurons in adult GAERS [16, 27], our study provides the first quantitative map of the maturation trajectory of PV+ and SST+ interneurons that are significantly altered during absence epileptogenesis in the GAERS model. Our findings fill a critical gap in knowledge by providing the first evidence for distinct spatiotemporal trajectories of PV+ and SST+ interneurons that may shape absence seizure epileptogenesis. Specifically, because PV+ interneurons are essential for fast FFI, their transient increase at P14 in S1 and M1 may critically disturb thalamocortical excitatory‐inhibitory balance and trigger the hypersynchronous activity underlying SWDs. In contrast, SST+ interneurons become selectively increased in the superficial layers of the M1 in later stages of development at P21 and in adulthood, suggesting that SST+ cells may not be the primary drivers of SWD initiation but instead may represent a structural substrate that facilitates the maintenance and generalization of seizure activity. Their increased density in M1 coincides with the maturation and generalization of SWDs that may further compromise inhibitory control, amplifying network susceptibility to absence seizures. Thus, PV+ interneurons may act as initiators of pathological oscillations, whereas SST+ interneurons function as modulators and propagators, sustaining epileptic activity once established. These findings led us to outline a hypothetical two‐phase framework in which PV+ and SST+ interneuron populations may be differentially associated with early and later phases of absence seizure activity (See graphical abstract). In this conceptual model, the PV+ and SST+ interneuron interaction and the thalamocortical circuitry, including the TRN, are illustrated based on previously established circuit organization and developmental maturation rather than observations made in the present study. Recent evidence further demonstrates that the TRN undergoes substantial postnatal functional maturation during the developmental period corresponding to the emergence of absence seizures, supporting its inclusion as a key component of the conceptual framework [28]. However, because the present study relies on immunohistochemical measures of interneuron density, the proposed framework should be regarded as a conceptual model rather than evidence of direct causal mechanisms.

Although thalamocortical electrophysiological dynamics have been extensively studied, cortical inhibitory neurodevelopment has received comparatively little attention. Rossignol et al. [7] showed that selective ablation of Cav2.1 channel function in cortical PV+ interneurons impaired GABA release and disinhibited pyramidal neurons, leading to absence and other generalized seizures, while other interneuron subtypes such as SST+ cells were unaffected. This is consistent with our observation that early PV+ interneuron alterations may play a pivotal role in initiating epileptiform activity, whereas SST+ interneurons appear less directly involved in seizure initiation. PV+ interneurons are fast‐spiking cells that primarily target the perisomatic region of pyramidal neurons and play a key role in controlling spike timing and network synchronization in cortical circuits [29]. In contrast, SST+ interneurons primarily target distal dendrites of pyramidal neurons and regulate dendritic integration of excitatory inputs, thereby shaping sustained network activity [30]. Within thalamocortical circuits implicated in absence epilepsy, such complementary inhibitory mechanisms could theoretically contribute to distinct phases of absence seizure dynamics.

Recent evidence demonstrates that pyramidal neurons are not passive targets of inhibition but act as active sculptors of inhibitory circuits, promoting the survival and terminal differentiation of specific interneuron subtypes to ensure proportionate coupling [31]. In light of this evidence, the altered PV+ and SST+ densities we observed in GAERS likely represent a non‐autonomous breakdown in this developmental matching process, creating a permissive environment vulnerable to hypersynchrony. Plutino et al. [12] demonstrated that cortical development in GAERS lacks the transient deep‐to‐superficial innervation normally present at P14 while simultaneously receiving prematurely enhanced thalamic drive at P15 [32]. This combination results in hyperexcitable pyramidal neurons and accelerates cortical circuit formation during a period typically dominated by sensory‐driven refinement. Our findings extend these observations by showing that, at the same developmental stage (P14), PV+ interneurons are markedly altered in GAERS, whereas SST+ interneurons remain unaffected. This suggests that early thalamocortical overdrive aligns with an early upregulation of PV+ interneurons, thereby strengthening FFI control during a critical maturation window. Since interneuron migration should be largely completed before the onset of SWDs [33], the observed increase in PV+ interneuron density is unlikely to reflect migratory defects. Instead, it may represent a compensatory attempt to counteract disrupted connectivity, which ultimately fails and may even indirectly contribute to SWD initiation by promoting pro‐epileptogenic high‐frequency oscillations [34]. At P25, GAERS display a significant increase in cortical GABA levels [20], which parallels the elevated PV+ interneuron population we observed at the same developmental stage. This suggests that enhanced PV‐mediated inhibition may underlie, at least in part, the rise in cortical GABAergic tone. Although initially compensatory, such excessive inhibition during a critical neurodevelopmental window may disrupt network maturation and FFI, thereby predisposing cortical circuits to maladaptive reorganization underlying absence epilepsy.

Our data indicate that these developmental alterations in PV+ expression are not limited to the cortex but also extend to hippocampal and striatal networks, highlighting a broader involvement of inhibitory circuit maturation in absence epileptogenesis. Interestingly, previous work has shown that by P21, newly generated neurons in the DG of GAERS, identified by doublecortin (a microtubule‐associated protein commonly used as a marker for immature neurons), exhibit increased synaptic integration with GABAergic terminals compared to Wistar rats [35]. Together, these findings suggest that the DG represents a region where inhibitory circuits undergo substantial remodeling during development, potentially influencing the trajectory of epileptogenesis in GAERS. Notably, this early alteration was not restricted to the DG; CA1 also showed a significant, transient increase in PV+ interneuron density at P14 in GAERS, indicating that the early disruption of hippocampal inhibitory circuit maturation extends across multiple hippocampal subfields rather than being confined to the DG. Although the hippocampus is not considered the primary locus of absence seizure initiation, such developmental deviations may indirectly influence network dynamics and contribute to the broader susceptibility of cortical and thalamic circuits to absence seizures.

PV+ interneurons, although sparse in number, exert powerful FFI on medium spiny neurons and are critical for controlling striatal output [36, 37]. In the GAERS model, striatal output neurons were shown to stop firing during ictal periods and display rebound activity afterwards, suggesting that disrupted striatal activity contributes to seizure propagation [38]. Miyamoto et al. [23] demonstrated that haplodeficiencies in *Stxbp1* and *Scn2a* (genes linked to human epilepsies) induce SWDs through reduced excitatory transmission from neocortex to striatal fast‐spiking interneurons, rather than via cortico‐thalamic circuits. Interestingly, we found that GAERS exhibit an increased density of striatal PV+ interneurons beginning at P21 and persisting into adulthood. This suggests that, despite a quantitative expansion of this inhibitory population, a reduction in effective cortical excitatory drive may compromise their functional recruitment. Such a mismatch between interneuron number and excitatory drive could represent a maladaptive form of developmental compensation, ultimately favoring aberrant synchronization within basal ganglia–thalamocortical loops. Notably, Miyamoto et al. [23] further showed that potentiating striatal ionotropic glutamate receptors (AMPA receptors) suppresses SWDs, supporting the idea that enhancing excitatory engagement of striatal PV+ interneurons might represent a promising therapeutic avenue in absence epilepsy. Our finding of elevated striatal PV+ interneurons suggests that developmental reorganization of this inhibitory microcircuit may constitute a network‐level adaptation that facilitates the propagation or maintenance of absence seizures.

SST+ interneurons contribute to processes associated with early circuit development including synaptogenesis, sensory innervation and neuronal maturation [39]. SST+ interneurons critically link sensory inputs with local circuits, regulating the developmental decorrelation of network dynamics in the developing cortex while modulating the maturation of PV+ interneurons [40]. Animal studies have shown that genetic ablation of *Cav2.1* channels in both PV+ and SST+ cortical interneurons elicits severe generalized seizures and absence seizures [7]. However, whether and how SST+ interneurons may contribute to absence seizure generation remains to be established. Functional studies have shown that SST+ interneurons in S1 are preferentially active during quiet wakefulness, the brain state where SWDs predominantly occur [41], and that their silencing increases bursting in cortical pyramidal neurons [42]. These observations highlight the state‐dependent contribution of SST+ circuits to cortical excitability. Considering our data, cortical SST+ interneurons showed no significant changes in the S1 across development but were increased in superficial layers of M1 at later stages (P21 and adulthood), pointing to region‐ and layer‐specific effects that may contribute to seizure maintenance rather than initiation. This increase in SST+ expression in layers II–IV of M1 may represent a compensatory response to enhanced excitatory drive from thalamic and intracortical inputs as absence seizures develop [43]. Recent work has highlighted a transient role for thalamo‐recipient SST+ interneurons in early cortical wiring, forming reciprocal connections with layer IV spiny stellate neurons prior to the maturation of PV+ mediated FFI [44]. This suggests that SST+ interneurons may serve an early, temporary scaffold during cortical assembly in some areas. However, they undergo later, region‐restricted remodeling. According to the work of Wu et al. [31], SST+ abundance is primarily regulated via programmed cell death (apoptosis) controlled by pyramidal neuron‐derived survival signals. Therefore, the late stage increase in SST+ density in the M1 of GAERS likely reflects a failure in the normal pruning process, where an altered cortical environment promotes excessive SST+ survival. In other words, SST+ circuits appear relatively preserved during the initial, PV‐dominated phase of epileptogenesis but may be recruited or expanded during later stages in selected cortical loci thereby creating a permissive structural environment that complements early initiation mechanisms.

Beyond the cortex, our results showed significantly increased striatal SST+ interneuron populations in adult GAERS. Although PV+ exerts strong proximal inhibition on striatal projection neurons, SST+ interneurons provide more distal and long‐range inhibitory inputs not only to striatal projection neurons but also to cholinergic interneurons. Thus, an increased SST+ interneuron population may alter both direct inhibitory control over striatum and the modulatory influence exerted via cholinergic interneurons. Such dual effects could contribute to maladaptive striatal reorganization in absence epilepsy, ultimately shaping basal ganglia–thalamocortical dynamics.

PV+ and SST+ interneurons provide complementary inhibition, perisomatic versus dendritic, crucial for the temporal and spatial organization of neuronal firing. An imbalance or developmental desynchronization between these subpopulations may disrupt excitatory input integration and oscillatory dynamics. The transition from synchronous to decorrelated activity in the cerebral cortex involves a developmental shift in the role of early SST+ and PV+ interneuron activity [39, 40, 44]. Interestingly, recent data demonstrate that SST+ interneurons promote the electrophysiological maturation of PV+ interneurons in response to sensory input [45], implying that disrupted SST–PV developmental interactions may underlie the premature emergence of PV+ interneurons observed in GAERS. Such a shift in inhibitory maturation timing could create an imbalance in cortical microcircuits, predisposing to the hypersynchronous network activity that culminates in absence seizures.

## 5 Conclusion

To date, no studies have addressed how these two interneuron subtypes interact during the early stages of epileptogenesis in genetic absence epilepsy models. Our data suggest that this developmental dissociation between SST+ and PV+ interneurons may underpin the network instability required for SWD generation, pointing to a critical gap. Whether the observed alterations in PV+ and SST+ densities in GAERS result from aberrant early migration or are a consequence of enhanced electrophysiological activity during critical developmental windows remains to be established. However, our proposed two‐phase model provides a testable framework for future functional studies. To move beyond structural correlations, specific circuit‐level or cell type‐specific interventions, using optogenetics and/or chemogenetic tools as well as pharmacological modulation of interneurons during the critical time windows for absence epileptogenesis, could confirm their distinct roles in seizure initiation and maintenance. Such circuit‐level investigations will be essential to validate or refine the two‐phase model proposed here.

## 6 Study Limitations

We acknowledge that the present study relies exclusively on immunohistochemical quantification of interneuron populations, which provides information on their distribution and density but does not establish causal relationships or directly assess synaptic function, neuronal firing properties, or network dynamics.

## Author Contributions

Study design: N.Ç., D.K., F.O., M.A. Data generation: E.T.E, N.Ç., M.A. Data analysis and interpretation: N.Ç., E.T.E., M.A., F.O., D.K. Manuscript preparation: N.Ç., E.T.E., D.K., F.O., M.A. Manuscript editing and critical revisions to the manuscript: N.Ç., D.K., F.O., M.A. All authors reviewed the manuscript.

## Funding

This work was funded by the European Commission Horizon Europe Programme under the call HORIZON‐WIDERA‐2021‐ACCESS‐03 [Grant Number 101078981‐GEMSTONE]. ETE is a TÜBİTAK 2214‐A and YÖK 100/2000 PhD scholar.

## Ethics Statement

All animal experiments were conducted in accordance with relevant guidelines and regulations and were approved by the Institutional Animal Care and Use Committee of Acıbadem University (Approval No: 2022/82).

## Conflicts of Interest

The authors declare no conflicts of interest.

## Supporting information


**Table S1:** Main effects and Strain × Age interaction from linear mixed models fit separately for each brain region and cell type (16 panels), with animal as a random intercept. Residual variance was allowed to differ by age where a likelihood ratio test indicated significant heteroscedasticity (“Age‐hetero variance” column).
**Table S2:** Pairwise strain comparisons (GAERS−Wistar) at each age within each region × cell type panel, with Benjamini–Hochberg FDR correction applied across all 48 comparisons.

## Data Availability

The data that support the findings of this study are available from the corresponding author upon reasonable request.
