# Supplementary material for: Neurodevelopmental Alterations in Inhibitory Interneurons of Cortex, Hippocampus, and Striatum of Genetic Absence Epilepsy Rats
Source: CNS Neurosci Ther. 2026 Sep 3;32(9):e71110. doi: 10.1002/cns.71110 (PMC13540994; doi:10.1002/cns.71110)
Supplement: Supplementary file 1 [file CNS-32-e71110-s001.docx]

***Table S1.*** *Main effects and Strain × Age interaction from linear mixed models fit separately for each brain region and cell type (16 panels), with animal as a random intercept. Residual variance was allowed to differ by age where a likelihood ratio test indicated significant heteroscedasticity (“Age-hetero variance” column).*

| **Region** | **Cell type** | **Strain** | **p** | **Age** | **p** | **Strain × Age** | **p** | **Age-hetero variance** |
| --- | --- | --- | --- | --- | --- | --- | --- | --- |
| S1 L2/4 | PV | F(1,30)=12.79 | .001** | F(2,30)=12.88 | <.001*** | F(2,30)=1.26 | .298 | Yes |
| S1 L2/4 | SST | F(1,30)=0.02 | .891 | F(2,30)=11.59 | <.001*** | F(2,30)=0.87 | .428 | Yes |
| S1 L5/6 | PV | F(1,30)=8.52 | .007** | F(2,30)=41.91 | <.001*** | F(2,30)=0.24 | .790 | No |
| S1 L5/6 | SST | F(1,30)=1.96 | .172 | F(2,30)=40.20 | <.001*** | F(2,30)=0.84 | .443 | Yes |
| M1 L2/4 | PV | F(1,30)=17.04 | <.001*** | F(2,30)=45.83 | <.001*** | F(2,30)=0.37 | .697 | Yes |
| M1 L2/4 | SST | F(1,30)=3.34 | .078 | F(2,30)=6.47 | .005** | F(2,30)=0.83 | .447 | No |
| M1 L5/6 | PV | F(1,30)=7.13 | .012* | F(2,30)=15.18 | <.001*** | F(2,30)=0.79 | .464 | No |
| M1 L5/6 | SST | F(1,30)=0.18 | .672 | F(2,30)=29.19 | <.001*** | F(2,30)=1.64 | .211 | No |
| CPU (Striatum) | PV | F(1,30)=5.40 | .027* | F(2,30)=14.64 | <.001*** | F(2,30)=1.17 | .326 | Yes |
| CPU (Striatum) | SST | F(1,30)=1.24 | .273 | F(2,30)=14.31 | <.001*** | F(2,30)=2.19 | .130 | No |
| DG | PV | F(1,30)=64.96 | <.001*** | F(2,30)=67.00 | <.001*** | F(2,30)=25.43 | <.001*** | Yes |
| DG | SST | F(1,30)=1.31 | .261 | F(2,30)=25.88 | <.001*** | F(2,30)=0.50 | .612 | Yes |
| CA1 | PV | F(1,30)=9.17 | .005** | F(2,30)=41.39 | <.001*** | F(2,30)=3.38 | .047* | Yes |
| CA1 | SST | F(1,30)=1.10 | .302 | F(2,30)=14.08 | <.001*** | F(2,30)=1.34 | .277 | No |
| CA3 | PV | F(1,30)=2.11 | .157 | F(2,30)=40.81 | <.001*** | F(2,30)=1.82 | .179 | Yes |
| CA3 | SST | F(1,30)=0.92 | .345 | F(2,30)=25.85 | <.001*** | F(2,30)=0.38 | .684 | Yes |

*Note. Density (cells/mm²) was averaged across 3 technical replicate sections per animal prior to analysis (n = 6 animals/strain/age). *** p < .001, ** p < .01, * p < .05.*

***Table S2.*** *Pairwise Strain comparisons (GAERS − Wistar) at each Age within each Region × Cell type panel, with Benjamini–Hochberg FDR correction applied across all 48 comparisons.*

| **Region** | **Cell type** | **Age** | **Estimate (GAERS−Wistar)** | **SE** | **df** | **p (raw)** | **p (FDR)** |
| --- | --- | --- | --- | --- | --- | --- | --- |
| S1 L2/4 | PV | P14 | 64.67 | 18.08 | 30 | .001 | .010** |
| S1 L2/4 | PV | P21 | 41.42 | 18.44 | 30 | .032 | .103 |
| S1 L2/4 | PV | Adult | 25.31 | 17.02 | 30 | .147 | .318 |
| S1 L2/4 | SST | P14 | -1.41 | 10.18 | 30 | .891 | .910 |
| S1 L2/4 | SST | P21 | 15.68 | 8.43 | 30 | .073 | .196 |
| S1 L2/4 | SST | Adult | 11.55 | 8.12 | 30 | .165 | .318 |
| S1 L5/6 | PV | P14 | 24.39 | 8.36 | 30 | .007 | .035* |
| S1 L5/6 | PV | P21 | 19.22 | 8.36 | 30 | .029 | .098 |
| S1 L5/6 | PV | Adult | 16.07 | 9.01 | 30 | .084 | .203 |
| S1 L5/6 | SST | P14 | -12.88 | 9.21 | 30 | .172 | .318 |
| S1 L5/6 | SST | P21 | -6.73 | 11.63 | 30 | .567 | .714 |
| S1 L5/6 | SST | Adult | 2.92 | 8.24 | 30 | .726 | .792 |
| M1 L2/4 | PV | P14 | 37.21 | 9.01 | 30 | <.001 | .005** |
| M1 L2/4 | PV | P21 | 27.71 | 11.81 | 30 | .026 | .098 |
| M1 L2/4 | PV | Adult | 25.02 | 13.52 | 30 | .074 | .196 |
| M1 L2/4 | SST | P14 | 19.61 | 10.74 | 30 | .078 | .196 |
| M1 L2/4 | SST | P21 | 29.79 | 10.74 | 30 | .009 | .045* |
| M1 L2/4 | SST | Adult | 39.14 | 10.74 | 30 | .001 | .010** |
| M1 L5/6 | PV | P14 | 27.76 | 10.40 | 30 | .012 | .053 |
| M1 L5/6 | PV | P21 | 14.95 | 10.45 | 30 | .163 | .318 |
| M1 L5/6 | PV | Adult | 9.83 | 10.40 | 30 | .352 | .497 |
| M1 L5/6 | SST | P14 | -4.35 | 10.17 | 30 | .672 | .768 |
| M1 L5/6 | SST | P21 | -5.60 | 10.17 | 30 | .586 | .714 |
| M1 L5/6 | SST | Adult | 17.57 | 10.17 | 30 | .094 | .216 |
| CPU (Striatum) | PV | P14 | 6.01 | 2.59 | 30 | .027 | .098 |
| CPU (Striatum) | PV | P21 | 10.11 | 2.54 | 30 | <.001 | .005** |
| CPU (Striatum) | PV | Adult | 12.26 | 3.64 | 30 | .002 | .014* |
| CPU (Striatum) | SST | P14 | 3.64 | 3.27 | 30 | .273 | .437 |
| CPU (Striatum) | SST | P21 | 6.57 | 3.27 | 30 | .053 | .160 |
| CPU (Striatum) | SST | Adult | 13.08 | 3.27 | 30 | <.001 | .005** |
| DG | PV | P14 | 33.27 | 4.13 | 30 | <.001 | <.001*** |
| DG | PV | P21 | 1.65 | 4.24 | 30 | .699 | .781 |
| DG | PV | Adult | -1.53 | 2.86 | 30 | .595 | .714 |
| DG | SST | P14 | 13.98 | 12.20 | 30 | .261 | .431 |
| DG | SST | P21 | -4.06 | 13.40 | 30 | .764 | .815 |
| DG | SST | Adult | 6.87 | 9.93 | 30 | .494 | .641 |
| CA1 | PV | P14 | 25.86 | 8.54 | 30 | .005 | .030* |
| CA1 | PV | P21 | 5.84 | 7.62 | 30 | .449 | .599 |
| CA1 | PV | Adult | -3.41 | 7.51 | 30 | .653 | .764 |
| CA1 | SST | P14 | -8.82 | 8.39 | 30 | .302 | .467 |
| CA1 | SST | P21 | -0.12 | 8.39 | 30 | .989 | .989 |
| CA1 | SST | Adult | 10.60 | 8.39 | 30 | .216 | .371 |
| CA3 | PV | P14 | 9.48 | 6.53 | 30 | .157 | .318 |
| CA3 | PV | P21 | 7.25 | 5.45 | 30 | .194 | .344 |
| CA3 | PV | Adult | -3.61 | 4.61 | 30 | .440 | .599 |
| CA3 | SST | P14 | 11.45 | 11.92 | 30 | .345 | .497 |
| CA3 | SST | P21 | -1.94 | 12.08 | 30 | .873 | .910 |
| CA3 | SST | Adult | 9.96 | 10.27 | 30 | .340 | .497 |

*Note. Estimate = mean difference in cell density (GAERS − Wistar), cells/mm². p (raw) = uncorrected p-value from the model-based simple-effect contrast. p (FDR) = Benjamini–Hochberg-adjusted p-value across all 48 comparisons. *** p < .001, ** p < .01, * p < .05 (FDR-adjusted).*
